# Anxiety Is a Mediator between Heart Rate Variability and Quality of Life in Chronic Obstructive Pulmonary Disease

**DOI:** 10.3390/jpm12060960

**Published:** 2022-06-12

**Authors:** Da-Wei Wu, Li-Hsin Chang, Po-Chou Yang, Tzu-Yu Kuo, Dong-Lin Tsai, Huang-Chi Chen, Hui-Lan Yuan, Pei-Shih Chen, Szu-Chia Chen, I-Mei Lin

**Affiliations:** 1 Doctoral Degree Program, Department of Public Health, College of Health Sciences, Kaohsiung Medical University, Kaohsiung 807, Taiwan; u8900030@yahoo.com.tw; 2 Department of Internal Medicine, Kaohsiung Municipal Siaogang Hospital, Kaohsiung Medical University, Kaohsiung 812, Taiwan; amorfati999@gmail.com (T.-Y.K.); huangchichen@gmail.com (H.-C.C.); 860021@kmuh.org.tw (H.-L.Y.); scarchenone@yahoo.com.tw (S.-C.C.); 3 Division of Pulmonary and Critical Care Medicine, Department of Internal Medicine, Kaohsiung Medical University Hospital, Kaohsiung Medical University, Kaohsiung 807, Taiwan; 4 Research Center for Environmental Medicine, Kaohsiung Medical University, Kaohsiung 807, Taiwan; pschen@kmu.edu.tw; 5 Department of Psychology, College of Humanities and Social Sciences, Kaohsiung Medical University, Kaohsiung 807, Taiwan; olivia1999hsin@gmail.com (L.-H.C.); leelo02227@gmail.com (P.-C.Y.); 6 Department of Surgery, Kaohsiung Municipal Siaogang Hospital, Kaohsiung Medical University, Kaohsiung 812, Taiwan; anakin711112@gmail.com; 7 Department of Public Health, College of Health Sciences, Kaohsiung Medical University, Kaohsiung 807, Taiwan; 8 Institute of Environmental Engineering, College of Engineering, National Sun Yat-Sen University, Kaohsiung 804, Taiwan; 9 Department of Medical Research, Kaohsiung Medical University Hospital, Kaohsiung 807, Taiwan; 10 Division of Nephrology, Department of Internal Medicine, Kaohsiung Medical University Hospital, Kaohsiung Medical University, Kaohsiung 807, Taiwan; 11 Faculty of Medicine, College of Medicine, Kaohsiung Medical University, Kaohsiung 807, Taiwan

**Keywords:** anxiety, chronic obstructive pulmonary disease, heart rate variability, quality of life

## Abstract

Autonomic nervous system (ANS) dysregulation is an important pathophysiological mechanism in patients with chronic obstructive pulmonary disease (COPD). Heart rate variability (HRV) is a common index for ANS, and HRV has been used to explore the association between ANS and clinical illnesses. This study aimed to explore the group differences in HRV, depression, anxiety, and quality of life between participants with COPD and healthy controls (HC group), and whether emotion plays a mediating role between HRV and quality of life in participants with COPD. A total of ninety-six participants with COPD and 59 participants in the HC group completed the Beck Depression Inventory-II (BDI-II), Beck Anxiety Inventory (BAI), and Saint George’s Respiratory Questionnaire (SGRQ). Assessment of spirometry pulmonary function and five minute lead II electrocardiography (ECG) were also performed under the resting baseline. The COPD group had higher depression scores (*F* = 4.10, *p* = 0.008), and a lower quality of life (*F* = 14.44, *p* < 0.001) and HRV indices (such as standard deviation of RR intervals (*F* = 5.49, *p* < 0.05) and low frequency (*F* = 3.03, *p* < 0.05)) compared to the HC group. Sympathetic activation was positively correlated with depression (*r* = 0.312, *p* < 0.01), anxiety (*r* = 0.420, *p* < 0.001), and poor quality of life (*r* = 0.467, *p* < 0.001) in the COPD group. After controlling for age and sex, anxiety (*β* = 0.585, *p* < 0.001) and sympathetic activation (*β* = 0.231, *p* < 0.05) positively predicted poor quality of life, and lung function (*β* = −0.251, *p* < 0.01) negatively predicted poor quality of life. Therefore, anxiety is a mediator between sympathetic activation and quality of life. Emotional and HRV screening should be applied to COPD patients in clinical practice, and emotional management or HRV biofeedback training can be used to improve anxiety and HRV for future studies.

## 1. Introduction

The Global Initiative for Chronic Obstructive Lung Disease states that chronic obstructive pulmonary disease (COPD) is caused by exposure to harmful particles, gases, smoke, and air pollutants, and is characterized by irreversible obstruction of expiratory airflow [1,2,3]. COPD is often associated with other diseases such as cardiovascular disease, hypertension, diabetes, tuberculosis, cancer, and stroke; and COPD can result in severe breathing difficulties, negative emotions, and poor quality of life [4].

Long-term smoking or exposure to air pollutants stimulates airway inflammation. The thickening and narrowing of the respiratory tract causes clinical symptoms such as gasping, chest tightness, and coughing, and causes a decline in lung function and lung gas exchange capacity, finally leading to disability and death [1,5,6,7]. In addition to inflammation and immune response, a systematic review found that patients with COPD have autonomic dysfunction [8], with a higher heart rate, lower heart rate variability (HRV), and respiratory sinus arrhythmia [9,10,11,12,13,14,15]. It may be due to the patient’s rapid and shallow breathing pattern, decreased sensitivity of baroreflex, and increased sensitivity of chemoreflex. These factors can then cause hyperactivity of the sympathetic nervous system (SNS), dysregulation of the SNS and parasympathetic nervous system (PNS) and worsen the symptoms of clinical dyspnea in participants with COPD [8,9].

HRV is commonly used as an index of autonomic nervous system (ANS) activation. Compared with healthy controls, COPD patients have been shown to have a lower time domain of HRV (standard deviation of RR intervals (SDNN) and root mean square of successive differences between normal heartbeats (RMSSD)) [10,11,13,14,15]. Some studies have also reported that COPD patients have a lower frequency domain of HRV (low frequency (LF) or high frequency (HF)) than healthy controls, reflecting lower overall HRV and PNS activity in COPD participants than in healthy controls [10,13,14,15,16]. Stein et al. [17] measured 24 h electrocardiography (ECG) and reported that daytime HRV indices (SDNN, RMSSD, natural logarithm of high frequency (lnHF) and natural logarithm of low frequency (lnLF)) in COPD patients were positively correlated with the percentage of predicted forced expiratory volume in the first second (FEV1/Predicted). Taken together, these findings suggest that lower HRV is related to more severe symptoms of COPD. 

Previous studies revealed that COPD patients have higher rates of depression and anxiety compared to the healthy population [18]. Despite the evidence showing a high prevalence and exceedingly negative impact of depression and anxiety in patients with COPD, depression and anxiety are rarely screened in clinical practice [18]. A systematic review and meta-analysis study confirmed the bidirectional associations between depression/anxiety and COPD and indicated that COPD in combination with depression and anxiety may worsen prognosis and increase the risk of mortality [19]. The severity of COPD has been found to be associated with ANS dysregulation, including lower overall HRV (SDNN) and PNS activation (RMSSD and natural logarithm of HF (lnHF)) [17], higher SNS activity (LF/HF ratio) [20], and poor quality of life [21]. However, few studies have explored the associations between emotion, ANS, and quality of life, and it is not clearly defined whether emotion and ANS may cause poor quality of life in participants with COPD. 

Therefore, this study aimed to examine the differences in depression, anxiety, HRV indices, and quality of life between the COPD and HC groups, and to explore whether emotion plays a mediating role between ANS regulation and quality of life in participants with COPD.

## 2. Materials and Methods

### 2.1. Participants 

The participants were referred by physicians at the Department of Thoracic Medicine and Health Management Center of Kaohsiung Municipal Siaogang Hospital and included 112 participants with COPD (COPD group) and 99 healthy controls (HC group). The inclusion criteria for the COPD group were physician-diagnosed COPD according to a ratio of forced expiratory volume in the first second (FEV1) to forced vital capacity (FVC) (FEV1/FVC ratio) < 70%, and post bronchodilation FEV1 increase below 12%. The inclusion criteria for the HC group were age above 37 years old that compared to age of COPD group, and no physical illnesses or mental disorders. The exclusion criteria in both groups were: (1) current or previous severe physical illness (such as asthma, respiratory failure, heart disease, cancer, etc.) or a mental disorder (such as substance use disorder, dementia, schizophrenia, bipolar disorder, etc.); (2) working shifts; (3) unstable vital signs (such as systolic blood pressure (SBP) ≥ 150 mmHg, heart rate ≥ 110 bpm, and SpO2 < 90%); and (4) participants with ECG artifacts or arrhythmia ≥ 5% in a 5 min measurement period. 

### 2.2. Measurements

Demographic data were recorded, and the following self-reported questionnaires were used. The Beck Depression Inventory-II (BDI-II) [22] was used to measure total depression score; the Beck Anxiety Inventory (BAI) [23] was used to measure total anxiety score; the Saint George’s Respiratory Questionnaire (SGRQ) [24] was used to measure respiratory quality of life. The Chinese versions of BDI-II [25,26], BAI [27,28], and SGRQ [29,30] were shown to have good psychometric properties. Blood pressure, ECG, and breathing rates were measured. Blood pressure was measured using a Blood Pressure Monitor (MW821fCA, Rossmax International Ltd., Taipei, Taiwan), and SBP and diastolic blood pressure (DBP) were recorded. The SpO2 was measured by a pulse oximeter which is a non-invasive device to monitor the amount of finger blood oxygen. An ECG sensor with a 2048 sampling rate was placed on the participants’ chest to obtain lead II ECG raw signals, and a respiration sensor was placed on participants’ chest to obtain the breathing rates. The raw physical signals were recorded by the ProComp Infiniti with BioGraph Infiniti version 6.0 (Thought Technology, Montreal, QC, USA). 

### 2.3. The Experimental Procedure

After finishing the psychological questionnaires, a researcher confirmed that the participants’ vital signs were in a stable state (SBP < 150 mmHg, heart rate < 110 bpm, and SpO2 ≥ 90%), and then lead II ECG and breathing rate were measured under sitting and resting baselines for 5 min. 

### 2.4. Data Processing and Statistical Analysis

The HRV Analysis Module (Thought Technology, Montreal, QC, USA) was used to transform the ECG to HRV indices, including (1) the time-domain of HRV including SDNN which refers to the total HRV; and (2) the frequency-domain of HRV including low-frequency power (LF, 0.04–0.15Hz; influenced by both sympathetic and parasympathetic activation or baroreceptor gain), high-frequency power (HF, 0.15–0.4 Hz; refers to parasympathetic activation), and low-frequency/high-frequency ratio (LF/HF ratio; refers to sympathetic activation). 

Data were analyzed using IBM SPSS Statistics for Windows, version 21.0 (IBM Corp., Armonk, NY, USA). The *t*-test and chi-square test were used to compare the group differences in demographic data (age and sex). The analysis of covariance (ANCOVA) was used to analyze the group differences in depression, anxiety, quality of life, spirometry, breathing rate, heart rate, SpO2, blood pressure, and HRV indices after controlling for age and sex. Pearson’s correlation analysis was used to examine correlations between depression, anxiety, quality of life, spirometry, and HRV indices in the COPD and HC groups. After controlling for age and sex, hierarchical regression analysis was used to examine the predictive ability of depression, anxiety, HRV indices, and spirometry for quality of life. The mediation effect models were used to examine depression and anxiety as mediators between HRV and quality of life after controlling for covariables.

## 3. Results

### 3.1. Participants’ Characteristics

A total of 112 participants with COPD were recruited, and 16 COPD participants were excluded from data analysis, due to ECG artifacts (*n* = 4), arrhythmia (*n* = 8), heart disease (*n* = 8), SBP ≥ 150 mmHg (*n* = 3), and did not finish the study procedure (*n* = 1). Finally, 96 COPD participants completed the study. For the HC group, 99 healthy participants were referred from the Health Management Center, and 65 were excluded from data analysis, which was due to comorbidities with other diseases (*n* = 24), absence of spirometry data (*n* = 24), high BDI-II or BAI scores (*n* = 10), ECG artifacts (*n* = 3), SBP ≥ 150 mmHg (*n* = 2), age < 24 years (*n* = 1), and not finishing the study procedure (*n* = 1). 

Finally, 34 healthy controls were included in the data analysis. The mean age was 66.25 ± 8.69 years (90% were males) for the COPD group, and 61.38 ± 7.56 years (47% were males) for the HC group (Table 1). There were significant differences in age (*t* = −2.90, *p* < 0.05) and sex (*x*^2^ = 21.07, *p* < 0.001).

### 3.2. Differences in Depression, Anxiety, Quality of Life, Spirometry, and HRV Indices between the HC and COPD Groups

After controlling for age and sex, the results of ANCOVA revealed higher depression and anxiety, poorer quality of life, and lower spirometry and SpO2 in the COPD group compared to the HC group (Table 1); as well as lower HRV indices in the COPD group compared to the HC group, including SDNN and LF (Table 1). Moreover, 10.42% of the COPD participants had at least mild depression (BDI-II score higher than 14), and 11.46% had at least mild anxiety (BAI score higher than 8).

### 3.3. Correlations between HRV Indices, Depression, Anxiety, Quality of Life, and Spirometry in Participants with COPD

Pearson’s correlation analysis revealed significantly positive correlations between LF/HF ratio and depression (r = 0.312, *p* < 0.01), LF/HF ratio and anxiety (r = 0.420, *p* < 0.001), and LF/HF ratio and total SGRQ score (r = 0.467, *p* < 0.001). In addition, SGRQ (respiratory quality of life) was positively correlated with depression (r = 0.370, *p* < 0.01) and anxiety (r = 0.676, *p* < 0.001) (Table 2). However, there was no significant correlation between spirometry, HRV indices, and SGRQ in the COPD group; as well as no significant correlation between HRV indices, depression, anxiety, quality of life, and spirometry in the HC group.

### 3.4. HRV Indices, Depression, Anxiety, and Spirometry in Predicting SGRQ

After controlling for age and sex, hierarchical regression analysis revealed that anxiety (*β* = 0.585, *p* < 0.001) and LF/HF ratio (*β* = 0.231, *p* = 0.017) positively predicted SGRQ, and that FEV1/predicted (*β* = −0.251, *p* = 0.004) negatively predicted SGRQ (Table 3).

### 3.5. Anxiety Was a Mediator between Sympathetic Activation and Quality of Life

**Figure 1:**
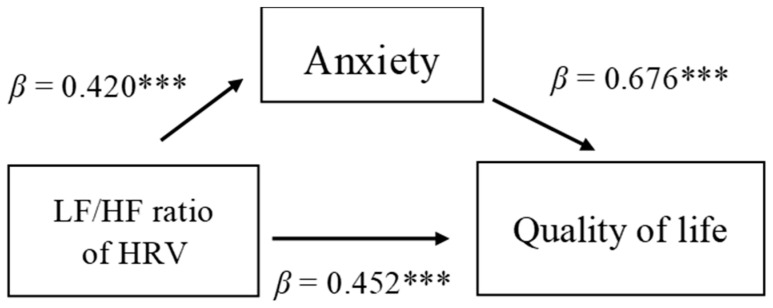
Mediation effect of anxiety between LF/HF ratio of HRV and quality of life. *** *p* < 0.001.

Mediation analysis revealed that anxiety was a mediator between LF/HF ratio and quality of life after controlling for age and sex. Model 4 demonstrated that anxiety was a positive predictor of quality of life (*β* = 0.533, *p* < 0.001) and LF/HF ratio was a positive predictor of quality of life (*β* = 0.228, *p* < 0.01), explaining 27.9% of the variation of quality of life (Table 4 and Figure 1).

## 4. Discussion

The most important findings of the present study were that the COPD group had higher depression and anxiety scores, and lower HRV indices and quality of life compared to the HC group. Anxiety and sympathetic activation were positive predictors of poor quality of life, and anxiety played an important mediating role between sympathetic activation and quality of life in COPD. 

The results of higher depression and anxiety and worse quality of life in COPD participants compared to the HC group are consistent with previous studies [20,31]. However, the prevalence of depression and anxiety in the COPD group was 10.42% and 11.46% respectively. The prevalence rates of depression and anxiety were slightly higher than the 4.1% for depression disorder [32] and 9.13% for anxiety disorder [33] reported in the analysis of the Taiwan National Health Insurance Database; but lower compared to the 27.1% and 17% reported for depression and anxiety scales in Western countries [34,35]; as well as 44.7% and 33.9% for prevalence of anxiety and depression in Tunisian patients with COPD [36]. Possible explanations for these findings are that depression and anxiety are not routinely screened in thoracic medicine clinics, or that the patients may not report their depression and anxiety symptoms to their doctors. Previous studies indicated that depression and anxiety may worsen the prognosis of COPD and increase mortality, such as non-compliance with clinical treatment, poor quality of life, increased hospitalization, length of hospital stays, and mortality [18,19,37,38].

Regarding ANS activation in COPD participants, the results are consistent with previous studies. Participants with COPD showed lower HRV indices compared to the HC group, including total HRV (SDNN) [10,11,13,14,15], SNS and PNS co-regulation (LF and lnLF) [10,12,13,14,15,16], and PNS index (HF of HRV) [10,13,14,15,16]. These findings reflect the ANS dysregulation in the COPD group compared to the HC group. In addition, this study also found that SNS activation (LF/HF ratio of HRV) was positively correlated with depression, anxiety, and poor quality of life in participants with COPD. This result is consistent with previous studies showing that higher negative emotions were related to poor quality of life [39,40,41], and higher SNS activation (LF/HF ratio) was related to poor quality of life [21]. However, Park et al. [42] did not find any association between SNS activation (LF/HF ratio) and quality of life (*r* = −0.027, *p* > 0.05). A possible reason may be because their study did not control for COPD comorbidities such as asthma, cancer, and cardiovascular disease. 

After controlling for age and sex, we found that anxiety and sympathetic activation (LF/HF ratio) significantly predicted quality of life, and FEV1/predicted significantly negatively predicted quality of life. This result is consistent with van Gestel et al. [21] who reported that higher parasympathetic activation (RMSSD) predicted better quality of life in COPD participants. There is a lack of studies examining the relationship between anxiety, HRV, and quality of life among patients with COPD. According to the mediation effect analysis suggested by Baron and Kenny [43], anxiety partly mediated the relationship between sympathetic activation (LF/HF ratio) and quality of life (SGRQ). Our results indicated that sympathetic activation affects patients’ quality of life through anxiety. Anxiety plays an important role in the relationship between sympathetic activation and quality of life in patients with COPD. Patients with high sympathetic activation and high levels of anxiety exhibit poor quality of life. Previous studies have confirmed the relationship between autonomic dysregulation and poor quality of life in patients with COPD [21,42]. Therefore, more attention should be paid to the emotional state of patients. In addition to appropriate medications, patients must also be given adequate psychological support.

There are several limitations in this study. First, there were significant differences in age and sex between the COPD group and the HC group. Although we tried to use matched case-control study designs, it is difficult to recruit middle-aged and elderly healthy adults in the hospital. We used ANCOVA to control the variances, but the influence of age and sex on the results cannot be ruled out. Second, only 34 participants have spirometry data in the HC group, because healthy participants do not routinely receive spirometry examinations in the health examination center. This may have affected comparisons between the two groups and the correlation analysis. Third, although this cross-sectional study finds that anxiety and sympathetic activation predicted poor quality of life, it cannot explain the causal relationship between these factors. A longitudinal study and follow-up study should be performed to examine the association between anxiety, sympathetic activation, and quality of life in further studies.

## 5. Conclusions

High levels of anxiety and sympathetic activation result in poor quality of life, and anxiety plays an important mediating role between sympathetic activation and poor quality of life. This study suggests that emotional screening in clinical practice, and psychological intervention, such as emotional management, mind-body exercise, or HRV biofeedback [44,45]. In future studies, these interventions may be applied to COPD rehabilitation programs to improve negative emotions and decrease sympathetic activation.

## Figures and Tables

**Table 1:** Demographic data and research variables between the COPD and HC groups.

	COPD Group (*n* = 96)	HC Group (*n* = 34)	*t/x* ^2^	*p*
Age, mean (years)	66.25 ± 8.69	61.38 ± 7.56	−2.90 **	0.004
Sex, male/female (%)	86/10 (90%/10%)	18/16 (53%/47%)	21.07 ***	<0.001
**ANCOVA ^a^**	**COPD group** **(*n* = 96)**	**HC group** **(*n* = 34)**	***F***	***p***
Total BDI-II score	5.35 ± 7.98	2.03 ± 2.43	4.10 **	0.008
Total BAI score	4.12 ± 7.52	1.33 ± 1.59	1.62	0.188
Total SGRQ score	20.29 ± 17.93	3.52 ± 5.36	8.40 ***	<0.001
Spirometry_FEV1(L)	1.64 ± 0.61	2.36 ± 0.51	32.15 ***	<0.001
FVC(L)	2.63 ± 0.77	2.85 ± 0.57	19.57 ***	<0.001
FEV1/Predicted (%)	59.02 ± 19.11	90.58 ± 17.31	61.52 ***	<0.001
FEV1/FVC (%)	61.77 ± 11.69	82.79 ± 9.32	59.69 ***	<0.001
Breathing rate (breaths/min)	17.63 ± 4.31	16.47 ± 3.65	1.51	0.222
Heart rate (beats/min)	76.05 ± 12.78	71.86 ± 8.77	1.28	0.284
SpO2 (%)	95.95 ± 1.70	96.65 ± 1.43	0.795	0.374
SBP (mmHg)	129.77 ± 13.42	125.76 ± 14.63	0.43	0.515
DBP (mmHg)	77.45 ± 10.84	76.74 ± 10.79	0.89	0.348
HRV_SDNN (ms)	21.17 ± 9.17	26.19 ± 9.51	5.49 *	0.021
LF (ms^2^/Hz)	28.19 ± 38.70	47.76 ± 52.95	3.03 *	0.032
HF (ms^2^/Hz)	41.86 ± 53.83	62.59 ± 64.21	2.86	0.093
LF/HF ratio	1.32 ± 1.44	1.26 ± 1.25	0.006	0.939

Data are expressed as (mean ± SD) unless otherwise indicated. * *p* < 0.05, ** *p* < 0.01, *** *p* < 0.001 ^a^ After controlling for age and sex, ANCOVA was used to compare the group differences in research variables. Abbreviations: BAI, Beck Anxiety Inventory; BDI-II, Beck Depression Inventory-II; COPD, chronic obstructive pulmonary disease; DBP, diastolic blood pressure; FEV1, forced expiratory volume in first second; FVC, forced vital capacity; HC, healthy controls; HF, high frequency; HRV, heart rate variability; LF, low frequency; LF/HF ratio, low frequency/high frequency ratio; SDNN, standard deviation of RR intervals; SBP, systolic blood pressure; SGRQ, Saint George’s Respiratory Questionnaire; SpO2, blood oxygen level.

**Table 2:** Correlations between HRV indices, anxiety, depression, spirometry, and quality of life in the COPD and HC groups.

	COPD (*n* = 96)					HC (*n* =34)				
	SDNN	LF	HF	LF/HF Ratio	SGRQ	SDNN	LF	HF	LF/HF Ratio	SGRQ
Total BDI-II score	−0.017	−0.038	−0.125	0.312 **	0.370 **	0.136	0.246	0.186	0.119	0.299
Total BAI score	−0.182	−0.033	−0.138	0.420 ***	0.676 ***	0.044	0.013	0.042	0.038	0.349
Total SGRQ score	−0.085	−0.075	−0.121	0.467 ***	1.00	−0.142	−0.104	−0.225	−0.235	1
Spirometry_FEV1	−0.061	0.094	−0.088	0.162	−0.171	0.101	−0.031	0.097	0.104	−0.065
FVC	−0.005	0.099	−0.048	0.134	−0.142	−0.026	−0.148	−0.054	−0.073	−0.083
FEV1/Predicted	−0.030	0.079	−0.018	0.052	−0.205	0.350 *	0.113	0.327	0.419 *	−0.203
FEV1/FVC	−0.145	0.023	−0.097	0.099	−0.168	0.255	0.263	0.291	0.322	0.010

* *p* < 0.05, ** *p* < 0.01, *** *p* < 0.001 Abbreviations: BAI, Beck Anxiety Inventory; BDI-II, Beck Depression Inventory-II; FEV1, forced expiratory volume in first second; FVC, forced vital capacity; HF, high frequency; HRV, heart rate variability; LF, low frequency; LF/HF ratio, low frequency/high frequency ratio; SDNN, standard deviation of RR intervals; SGRQ, Saint George’s Respiratory Questionnaire.

**Table 3:** Depression, anxiety, HRV indices, and spirometry in predicting quality of life for COPD (*n* = 96).

	Model 1			Model 2		
	*β*	*t*	*p*	*β*	*t*	*p*
Model 1						
Age	−0.097	−0.810	0.421	0.019	0.213	0.832
Sex	0.058	0.484	0.630	0.007	0.080	0.937
Model 2						
BDI-II				0.024	0.244	0.808
BAI				0.585 ***	5.925	<0.001
LF/HF ratio				0.231 *	2.445	0.017
FEV1/predicted				−0.251 **	−2.977	0.004
*R* ^2^		0.012			0.566 ***	
*F*		0.425			14.117	
*P*		0.656			<0.001	
Δ*R*^2^					0.554 ***	
Δ*F*					20.271	
Δ*P*					<0.001	

* *p* < 0.05, ** *p* < 0.01, *** *p* < 0.001 Abbreviations: BAI, Beck Anxiety Inventory; BDI-II, Beck Depression Inventory-II; FEV1, forced expiratory volume in first second; HRV, heart rate variability; LF/HF ratio, low frequency/high frequency ratio; Dependent variable: quality of life was measured by Saint George’s Respiratory Questionnaire (SGRQ).

**Table 4:** Mediation effect of anxiety between LF/HF ratio and quality of life for COPD (*n* = 96).

	BAI	SGRQ		
	Model 1	Model 2	Model 3	Model 4
LF/HF ratio	0.420 ***	0.452 ***		0.228 **
BAI			0.676 ***	0.533 ***
*R* ^2^	0.176	0.219	0.457	0.498
Δ*R*^2^				0.279
*F*	19.695 ***	19.752 ***	58.954 ***	34.234 ***

** *p* < 0.01, *** *p* < 0.001 Abbreviations: BAI, Beck Anxiety Inventory; LF/HF ratio, low frequency/high frequency ratio; SGRQ, Saint George’s Respiratory Questionnaire.

## Data Availability

Data can be shared by the corresponding author upon reasonable request.
